# Supplementary material for: Effects of Early Intervention with Maternal Fecal Microbiota and Antibiotics on the Gut Microbiota and Metabolite Profiles of Piglets
Source: Metabolites. 2018 Dec 6;8(4):89. doi: 10.3390/metabo8040089 (PMC6316024; doi:10.3390/metabo8040089)
Supplement: Supplementary file 1 [file metabolites-08-00089-s001.pdf]

Effects of early intervention with maternal fecal microbiota and antibiotics on the gut microbiota and metabolite profiles of piglets

Chunhui Lin, Jiajia Wan, Yong Su, Weiyun Zhu

College of Animal Science and Technology, Nanjing Agricultural University, Nanjing 210095, Jiangsu, China

National Center for International Research on Animal Gut Nutrition, Nanjing Agricultural University, Nanjing 210095, China

Corresponding author: Prof. Yong Su

Tel: 00 86 25 84395523; Fax: 00 86 25 84395314

E-mail: yong.su@njau.edu.cn

Table S1 Relative abundance (percentage) of microbial operational taxonomic units (OTUs) in the stomach of piglets in the microbiota transplantation (MFMT), amoxicillin treatment (AM), and control (CO) groups.

OTU ID	7d			21d			Annotation
	CO	AM	MFMT	CO	AM	MFMT	
OTU2	17.45±5.88[a]	3.36±1.84[b]	22.31±2.85[a]	18.09±1.14	18.89±1.5	13.74±3.05	g__*Lactobacillus*
OTU3	22.47±8.65	38.53±8.82	21.81±9.93	1.71±0.71	0.39±0.20	2.19±1.01	s__*Lactobacillus delbrueckii subsp. Sunkii*
OTU1	13.69±9.25	0.49±0.22	21.09±9.22	34.87±3.53	40.47±8.55	28.84±7.30	s__*Lactobacillus gallinarum*
OTU5	8.56±3.49[b]	23.91±5.30[a]	8.10±1.76[b]	0.15±0.06	0.13±0.05	0.08±0.05	g__*Lactobacillus*
OTU7	2.04±0.78	0.42±0.24[b]	2.62±0.40[a]	1.67±0.20	1.83±0.24	1.14±0.35	g__*Lactobacillus*
OTU4	4.64±3.01	4.93±3.03	2.61±1.18	23.26±7.10	22.91±7.82	25.55±5.11	g__*Lactobacillus*
OTU6	3.28±0.92[b]	14.54±2.91[a]	2.14±0.77[b]	0.15±0.05	0.15±0.06	0.21±0.11	s__*Lactobacillus mucosae*
OTU14	0.91±0.15	0.76±0.29	1.12±0.20	0.23±0.07	0.23±0.06	0.19±0.08	g__*Rothia*
OTU17	1.14±0.60	0.61±0.23	1.07±0.24	0.31±0.11	0.21±0.04	0.28±0.09	g__*Porphyromonas*
OTU13	1.40±0.66	0.72±0.43	1.04±0.25	0.26±0.08	0.19±0.04	0.20±0.08	g__*Moraxella*
OTU15	1.37±0.68	0.76±0.43	0.98±0.22	0.26±0.06	0.17±0.04	0.21±0.07	g__*Moraxella*
OTU18	0.79±0.22	0.63±0.24	0.81±0.14	0.56±0.28	0.23±0.04	0.24±0.08	s__*Streptococcus suis*
OTU8	4.01±2.12	0.19±0.10	0.79±0.37	0.24±0.04	0.49±0.21	0.63±0.29	g__*Lactobacillus*
OTU23	1.09±0.58	0.56±0.29	0.76±0.18	0.19±0.05	0.12±0.04	0.14±0.06	g__*Moraxella*
OTU16	0.87±0.43	0.41±0.14	0.71±0.25	0.59±0.36	0.41±0.12	0.39±0.11	s__*Actinobacillus porcinus*
OTU25	0.60±0.16	0.42±0.14	0.65±0.18	0.44±0.21	0.17±0.03	0.17±0.05	s__*Streptococcus suis*
OTU9	0.62±0.27	0.22±0.07	0.57±0.16	1.77±1.45	0.91±0.37	0.91±0.67	g__*Terrisporobacter*
OTU31	0.66±0.39	0.36±0.16	0.55±0.10	0.12±0.03	0.14±0.01	0.11±0.04	s__*Actinobacillus minor*
OTU27	0.72±0.55	0.20±0.13	0.51±0.10	0.44±0.16	0.31±0.04	0.28±0.10	s__*[Haemophilus] parasuis*
OTU11	0.32±0.18	0.10±0.05	0.44±0.18	1.01±0.11	1.09±0.09	0.98±0.09	g__*Lactobacillus*
OTU24	0.73±0.61	0.25±0.05	0.44±0.08	0.39±0.13	0.46±0.11	0.28±0.08	g__*Alloprevotella*
OTU19	2.13±2.04	0.68±0.15	0.35±0.19	0.07±0.01	0.14±0.05	0.39±0.31	g__*Veillonella*
OTU32	0.57±0.32	0.20±0.08	0.35±0.05	0.33±0.10	0.20±0.03	0.22±0.08	s__*[Haemophilus] parasuis*
OTU37	0.49±0.34	0.15±0.05	0.35±0.09	0.19±0.06	0.21±0.05	0.17±0.05	g__*Fusobacterium*
OTU38	0.64±0.38	0.20±0.12	0.30±0.06	0.04±0.01	0.05±0.01	0.05±0.02	g__*Leptotrichia*
OTU20	0.23±0.08	1.25±0.73	0.29±0.12	0.25±0.12	0.58±0.51	0.36±0.20	g__*Halomonas*
OTU44	0.26±0.14	0.09±0.03	0.29±0.05	0.10±0.04	0.10±0.02	0.13±0.07	g__*Helcococcus*
OTU22	0.29±0.10	0.09±0.02	0.27±0.08	0.92±0.76	0.52±0.21	0.42±0.30	g__*Clostridium sensu stricto 1*
OTU12	0.23±0.10	0.08±0.03	0.26±0.08	1.50±1.29	0.83±0.39	0.58±0.44	g__*Intestinibacter*
OTU43	0.36±0.31	0.22±0.17	0.25±0.07	0.07±0.03	0.07±0.03	0.12±0.06	g__*Streptobacillus*

[a, b] Mean values within a line with different superscript letters on each day differ significantly ($P < 0.05$).

Table S2 Relative abundance (percentage) of microbial operational taxonomic units (OTUs) in the ileum of piglets in the microbiota transplantatio (MFMT), amoxicillin treatment (AM), and control (CO) groups.

OTU ID	7d			21d			
	CO	AM	MFMT	CO	AM	MFMT	
OTU3	18.85±7.35	7.92±4.34	20.54±4.57	11.36±3.38	17.86±4.12	12.06±6.10	g__*Lactobacillus*
OTU1	13.20±8.15	1.17±0.84	19.19±7.86	36.27±6.84	33.72±7.23	20.10±7.13	s__*Lactobacillus gallinarum*
OTU6	10.35±6.18	18.09±3.33	15.34±8.60	0.60±0.18	0.15±0.11	1.74±1.08	s__*Lactobacillus delbrueckii subsp. sunkii*
OTU4	13.42±5.73	2.40±1.54	14.74±3.82	3.23±0.20	5.93±1.70^{a}	2.18±0.92^{b}	g__*Lactobacillus*
OTU5	9.52±4.11	22.12±5.77	10.93±3.17	0.02±0.01	0.02±0.01	0.06±0.04	g__*Lactobacillus*
OTU2	4.36±2.90	12.62±10.87	2.96±1.50	29.58±6.39	25.42±9.77	32.83±10.02	g__*Lactobacillus*
OTU10	1.36±0.81	2.92±0.34	2.73±1.56	0.14±0.05	0.04±0.04	0.28±0.17	g__*Lactobacillus*
OTU7	3.14±1.07^{b}	11.75±3.59^{a}	2.10±0.61^{b}	0.22±0.05	0.20±0.04	0.79±0.57	s__*Lactobacillus mucosae*
OTU20	0.79±0.47	1.47±0.29	1.18±0.67	0.05±0.01	0.02±0.01	0.13±0.08	s__*Lactobacillus delbrueckii subsp. sunkii*
OTU16	0.88±0.34	0.28±0.14^{b}	1.05±0.13^{a}	0.51±0.12	0.80±0.16	0.48±0.21	s__*Lactobacillus vaginalis*
OTU9	4.77±3.66	0.62±0.27	0.99±0.43	0.45±0.27	1.02±0.59	1.33±0.74	g__*Lactobacillus*
OTU12	0.56±0.34	0.06±0.05	0.96±0.39	1.71±0.31	1.73±0.37	1.15±0.47	g__*Lactobacillus*
OTU25	0.67±0.40	0.84±0.13	0.63±0.31	0.06±0.02	0.03±0.01^{b}	0.12±0.05^{a}	g__*Lactobacillus*
OTU28	0.48±0.14	0.15±0.12	0.45±0.04	0.14±0.04	0.46±0.18	0.19±0.08	s__*Lactobacillus vaginalis*
OTU15	1.44±1.21	0.94±0.69	0.40±0.18	0.72±0.35	1.49±1.31	0.15±0.07	g__*Intestinibacter*
OTU39	0.24±0.13	0.41±0.07	0.38±0.24	0.01±0.00	0.01±0.00	0.06±0.04	g__*Lactobacillus*
OTU24	0.35±0.22	0.00±0.00	0.37±0.15	0.47±0.10	0.68±0.19	0.33±0.13	g__*Lactobacillus*
OTU18	0.62±0.50	1.59±1.35	0.35±0.11	0.69±0.29	0.89±0.78	0.13±0.04	g__*Terrisporobacter*
OTU22	1.09±0.81	0.90±0.34	0.32±0.13	0.34±0.05	0.16±0.04	0.17±0.08	s__*Streptococcus suis*
OTU11	0.78±0.50	3.49±1.55^{a}	0.28±0.16^{b}	1.02±0.44	0.52±0.32	0.53±0.18	g__*Veillonella*
OTU19	0.21±0.09	0.20±0.17	0.27±0.11	1.01±0.04	0.90±0.08	0.84±0.13	g__*Lactobacillus*
OTU31	0.25±0.07^{a}	0.08±0.05^{b}	0.26±0.02^{a}	0.08±0.02^{b}	0.37±0.14^{a}	0.15±0.07	s__*Lactobacillus vaginalis*
OTU23	3.20±3.09	0.01±0.01	0.18±0.18	0.02±0.01	0.01±0.00	0.07±0.04	s__*Streptococcus macedonicus ACA-DC 198*
OTU8	0.17±0.07	0.03±0.01	0.15±0.04	5.40±2.53	3.49±1.49	5.53±3.10	s__*Lactobacillus panis*
OTU58	0.06±0.05	0.03±0.02	0.13±0.11	0.06±0.03	0.03±0.01	0.10±0.08	g__*Corynebacterium 1*
OTU47	0.17±0.11	0.15±0.04	0.13±0.07	0.05±0.01	0.03±0.02	0.07±0.03	g__*Peptostreptococcus*
OTU26	0.30±0.27	0.42±0.20	0.12±0.05	0.55±0.11	0.19±0.13	0.45±0.13	g__*Halomonas*
OTU30	2.08±2.00	0.05±0.03	0.11±0.10	0.07±0.04	0.01±0.01	0.05±0.02	g__*Streptococcus*
OTU37	0.49±0.40	0.34±0.08	0.09±0.06	0.08±0.04	0.02±0.01	0.13±0.08	g__*Fusobacterium*
OTU42	0.05±0.02	0.05±0.05	0.06±0.03	0.26±0.03^{a}	0.15±0.03^{b}	0.22±0.03	g__*Lactobacillus*

[a, b] Mean values within a line with different superscript letters on each day differ significantly ($P < 0.05$).

Table S3 Relative abundance (percentage) of microbial operational taxonomic units (OTUs) in the colon of piglets in the microbiota transplantation (MFMT), amoxicillin treatment (AM), and control (CO) groups.

OTU ID	7d			21d			Annotation
	CO	AM	MFMT	CO	AM	MFMT	
OTU1	5.62±1.70	0.87±0.55[b]	13.56±5.92[a]	2.84±1.58	6.63±2.79	5.72±3.80	g__*Lactobacillus*
OTU3	6.91±2.05	5.55±1.82	10.13±6.20	1.64±0.75	0.93±0.48	0.98±0.47	g__*Subdoligranulum*
OTU5	0.71±0.63[b]	0.12±0.11[b]	6.14±1.31[a]	0.93±0.39[b]	2.77±0.84	4.51±1.29[a]	f__*Bacteroidales S24-7 group*
OTU8	4.10±1.46	2.43±0.93	2.55±0.92	1.01±0.55	0.31±0.16	0.95±0.34	g__*Ruminococcus 2*
OTU10	0.63±0.45	1.76±0.73	2.43±1.90	0.87±0.42	2.90±1.59	1.41±0.47	g__*Collinsella*
OTU11	1.32±0.75	1.54±0.97	2.19±1.57	1.62±1.45	2.23±1.60	0.68±0.37	g__*Prevotella 2*
OTU17	0.99±0.46	0.26±0.20	1.98±1.35	3.03±2.61	1.08±0.42	0.42±0.26	g__*Prevotellaceae NK3B31 group*
OTU62	0.03±0.01	0.04±0.04	1.92±1.80	0.04±0.03	0.07±0.03	0.08±0.01	g__*Cloacibacillus*
OTU2	0.42±0.41	2.09±2.08	1.81±1.62	5.08±3.76	4.64±1.76	14.12±6.53	g__*Lactobacillus*
OTU40	0.00±0.00	1.51±1.27	1.26±1.26	0.12±0.08	0.62±0.39	0.05±0.04	g__*Alloprevotella*
OTU13	5.43±3.14	2.55±2.08	1.20±1.20	0.09±0.06	0.05±0.01	0.17±0.08	g__*Bacteroides*
OTU41	0.10±0.08[b]	0.62±0.31	1.13±0.45[a]	0.29±0.14	0.94±0.47	0.73±0.34	g__*Blautia*
OTU22	0.24±0.23	0.00±0.00	1.12±1.08	1.50±1.01	1.91±1.01	2.37±1.01	s__*Lactobacillus gallinarum*
OTU45	1.28±0.74	0.91±0.52	1.11±0.47	0.06±0.04	0.05±0.02	0.09±0.01	s__*Lactobacillus delbrueckii subsp. sunkii*
OTU19	0.03±0.02	0.90±0.77	1.08±0.74	1.74±0.61	1.62±0.49	1.84±0.63	g__*[Ruminococcus] gauvreauii group*
OTU46	0.18±0.12	0.46±0.12	1.04±1.01	0.31±0.20	0.34±0.20	0.83±0.32	g__*Escherichia-Shigella*
OTU30	3.85±3.70	0.18±0.15	0.93±0.78	0.25±0.20	0.35±0.20	0.53±0.28	g__*Fusobacterium*
OTU34	1.28±0.27	2.19±0.55[a]	0.86±0.25[b]	0.08±0.05	0.37±0.21	0.08±0.03	g__*Lachnoclostridium*
OTU27	0.01±0.00	2.82±2.57	0.77±0.48	0.65±0.34	1.23±0.82	0.57±0.21	g__*[Eubacterium] coprostanoligenes group*
OTU87	0.06±0.02	0.09±0.03	0.75±0.39	0.11±0.06	0.18±0.08	0.40±0.27	g__*Blautia*
OTU20	0.46±0.30	0.25±0.20	0.64±0.32	1.28±0.40	3.14±1.39	1.12±0.54	g__*Anaerotruncus*
OTU49	0.01±0.01	1.80±1.74	0.59±0.49	0.29±0.12	0.33±0.09	0.36±0.19	g__*Holdemanella*
OTU44	0.36±0.32	2.13±1.29	0.52±0.50	0.31±0.10	0.09±0.03	0.20±0.11	g__*Prevotellaceae UCG-004*
OTU26	0.11±0.06	0.03±0.01[b]	0.48±0.21[a]	1.86±0.83	2.74±0.59	1.00±0.35	s__*gut metagenome*
OTU23	1.35±1.03	1.58±1.02	0.48±0.23	1.19±1.10	0.76±0.50	1.03±0.79	g__*Methanobrevibacter*
OTU69	0.22±0.13	0.42±0.20	0.47±0.26	0.27±0.13	0.42±0.13	0.27±0.21	g__*Ruminococcaceae NK4A214 group*
OTU54	0.55±0.10	0.57±0.39	0.45±0.20	0.51±0.31	0.48±0.28	0.13±0.10	g__*Tyzzerella*
OTU56	0.10±0.03	0.34±0.27	0.44±0.21	0.70±0.31	0.37±0.16	0.76±0.25	g__*Lachnospiraceae FCS020 group*
OTU81	0.34±0.14	0.24±0.14	0.40±0.29	0.24±0.06	0.10±0.04[b]	0.44±0.16[a]	g__*Desulfovibrio*
OTU77	0.13±0.06	0.01±0.00[b]	0.39±0.16[a]	0.35±0.22	0.27±0.12	0.63±0.33	s__*Lactobacillus panis*

[a, b] Mean values within a line with different superscript letters on each day differ significantly ($P < 0.05$).
